# Fair Look at Coordination Oligomerization of Higher α-Olefins

**DOI:** 10.3390/polym12051082

**Published:** 2020-05-09

**Authors:** Ilya Nifant’ev, Pavel Ivchenko

**Affiliations:** A.V. Topchiev Institute of Petrochemical Synthesis RAS, 29 Leninsky Pr., 119991 Moscow, Russia

**Keywords:** dimerization, metallocenes, oils, oligomerization, single-site catalysts, polymerization

## Abstract

Coordination catalysis is a highly efficient alternative to more traditional acid catalysis in the oligomerization of α-olefins. The distinct advantage of transition metal-based catalysts is the structural homogeneity of the oligomers. Given the great diversity of the catalysts and option of varying the reaction conditions, a wide spectrum of processes can be implemented. In recent years, both methylenealkanes (vinylidene dimers of α-olefins) and structurally uniform oligomers with the desired degrees of polymerization have become available for later use in the synthesis of amphiphilic organic compounds and polymers, high-quality oils or lubricants, and other prospective materials. In the present review, we discussed the selective dimerization and oligomerization of α-olefins, catalyzed by metallocene and post-metallocene complexes, and explored the prospects for the further applications of the coordination α-olefin dimers and oligomers.

## 1. Introduction

**Scheme 1:**
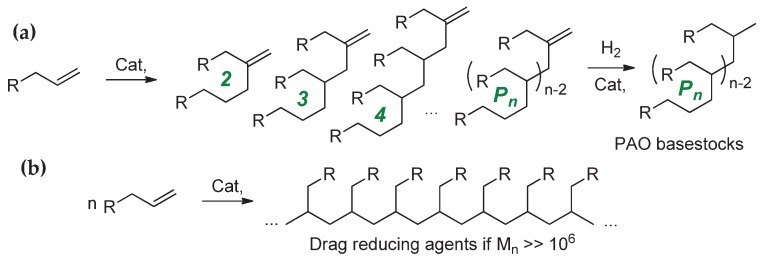
Coordination oligomerization (**a**) and polymerization (**b**) of α-olefins.

The achievement of the best characteristics of chemical product by the use of efficient knowledge-intensive technologies is the most productive avenue for achieving real development goals in the modern chemical industry. The Ziegler–Natta polymerization of α-olefins [1,2,3,4,5] is an excellent example of such technologies that provides humanity with ~2 × 10^8^ tons of plastics per year. Ethylene and propylene are raw materials for the modern polyolefin industry; thousands of articles and hundreds of reviews are devoted to the coordination homopolymerization and copolymerization of these monomers. The polymerization and oligomerization of higher α-olefins have been studied less intensively. The oligomerization of C_8_+ α-olefins followed by hydrogenation to form engine Group 4 poly-α-olefin oil (PAO) base stocks and lubricants (Scheme 1a) [6,7], and the synthesis of ultra-high MW polyolefins (Scheme 1b) as drag reducing agents [8,9], were actual topics of the applied research. 

It was the Group 4 oil industry that led to the growing interest in the oligomerization of higher α-olefins in the mid-20th century [10,11]. The conventional technologies of acid-catalyzed oligomerization with the use of BF_3_/ROH or Al chloride catalysts remains relevant for the production of the lower oligomers of α-olefins [12,13]. The cationic oligomerization is accompanied by huge numbers of rearrangements [14,15]. However, lower α-olefin oligomers obtained by cationic oligomerization were exceedingly defined as structurally uniform reaction products [16,17]. Moreover, most of the recent publications devoted to the oligomerization of higher α-olefins have discussed acid-catalyzed processes [18,19,20,21,22,23,24,25,26,27,28,29,30,31,32]. 

The review of Nicholas [7] was also focused on cationic polymerization; the mini-review of Ray et al., devoted to the synthesis of PAOs [12], addresses only part of the problem of coordination polymerization. The reviews of Janiak [33,34] and Belov [35] were focused on the coordination oligomerization of α-olefins, but these works, published more than 10 years ago, are now objectively outdated.

Taking into account the fact that the selective coordination oligomerization of α-olefins is a growth point of the actual petrochemical industry [7], in the present review, we tried to organize the current scientific information on that topic. In our review, we discussed the plausible mechanisms of the coordination dimerization and oligomerization of α-olefins, collated the data on the catalytic properties of different single-site catalysts, and discussed the problem of the catalyst design. Additionally, we were to focus on looking at the relationship between molecular structure and the characteristics of the oligomers of α-olefins, and on the prospects of the application of these dimers and oligomers in the synthesis of amphiphilic organic compounds and polymers, in the production of fuels, oils, lubricants, and other actual products.

## 2. Coordination Dimerization of α-Olefins

### 2.1. Group 4 Metallocene-Catalyzed Synthesis of Methylenealkanes

**Scheme 2:**
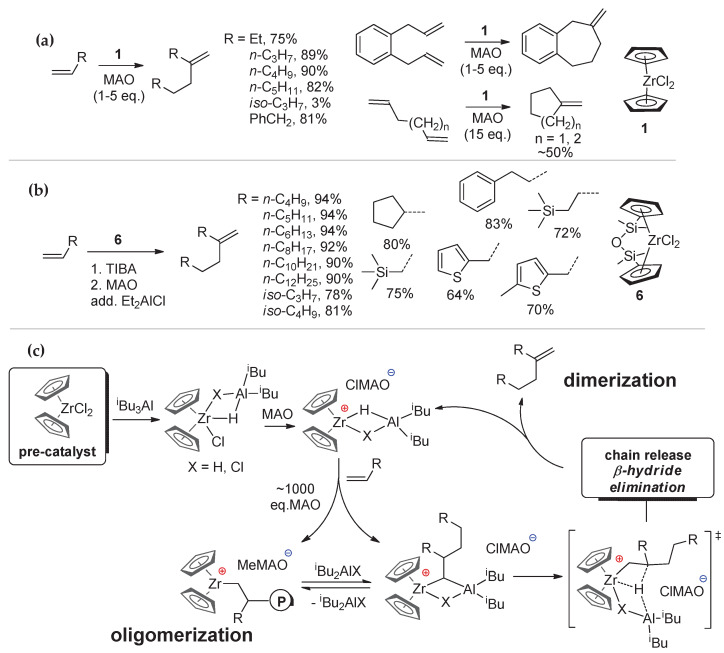
Early works on the zirconocene-catalyzed dimerization of α-olefins [37,38,39] (**a**); high efficiency of the pre-catalyst **6** [40,41] (**b**); cationic Zr–Al catalytic species proposed to explain the reactivity of zirconocene under low MAO/Zr ratios [40,41] (**c**).

The zirconocene-catalyzed dimerization of α-olefins (Scheme 2) has been known since the late 1980s [36]. The reaction proceeds in the presence of zirconocene dichloride (η^5^-C_5_H_5_)_2_ZrCl_2_ (**1**, Scheme 2a), activated by minimal amounts of methylalumoxane (MAO) and results in the selective formation of methylenealkanes, olefins containing the vinylidene fragment >C=CH_2_. The prospects of the synthetic use of this reaction in the synthesis of methylenealkanes were studied by Christoffers and Bergman for linear α-olefins, allylbenzene, and 1,2-diallylbenzene [37,38], and by Erker et al. for 1,5-hexadiene and 1,6-heptadiene [39] (Scheme 2a). The reaction was complicated by the isomerization of the starting α-olefins, by the formation of higher oligomers, and by the deactivation of the catalyst.

**Scheme 3:**
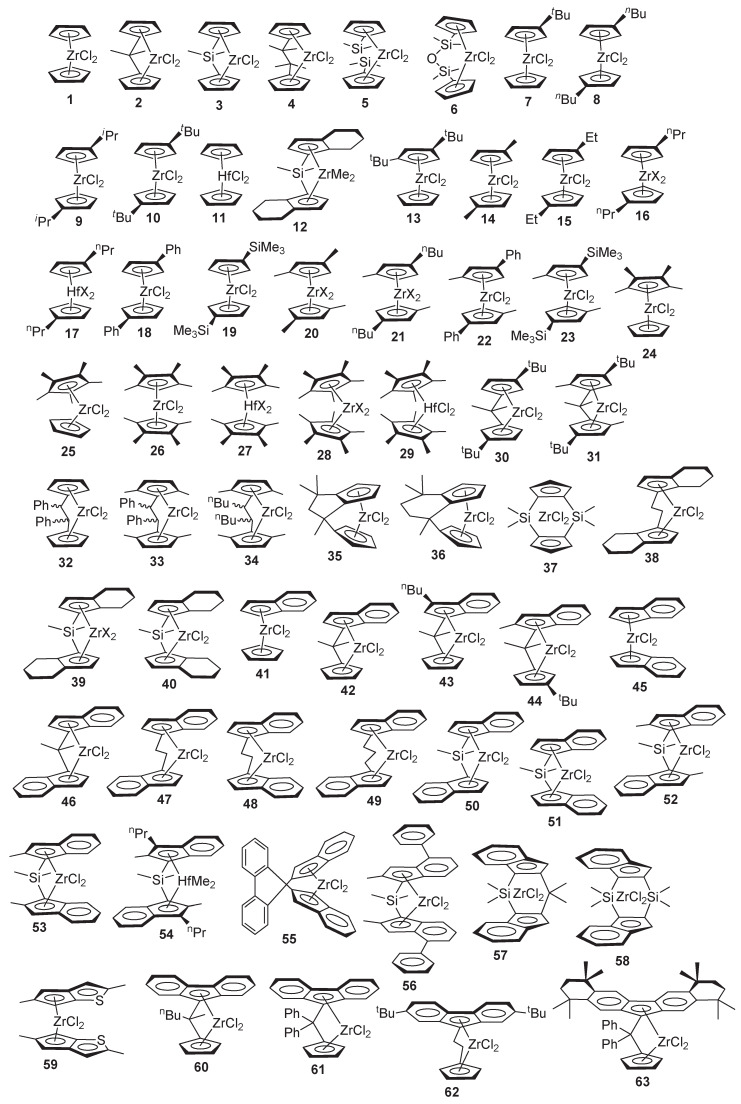
Metallocene pre-catalysts studied in the coordination oligomerization of α-olefins.

Nifant’ev et al. optimized this approach significantly. Based on the results of X-ray diffraction studies of bis(cyclopentadienyl) zirconium complexes **1**–**6** (Scheme 3), they proposed the structure of highly active and selective pre-catalyst **6**. The use of **6** activated by ~20 equivalents (eq.) of triisobutylalumnium (TIBA) and 10 eq. of methylalumoxane (MAO) provided 92%–94% yields of vinylidene dimers of linear α-olefins [40,41,42,43]. As compared with zirconocene **1**, in the presence of the **6** dimer of sterically hindered olefin, 3-methylbut-1-ene was obtained with an average yield; substituted silanes and allylthiophenes also formed vinylidene dimers [41] (Scheme 2b). Note that the zirconium pre-catalysts **7** [44,45,46], **8** [47], **3** [48], **9** [49,50], and **10** [51] and the hafnium complex **11** [48], studied previously in α-olefin dimerization, were less active and selective in comparison with **6**.

The replacement of the activator of the zirconocene pre-catalyst by perfluoroaryl borate resulted in the migration of the C=C bond: thus, in the presence of dimethyl zirconocene **12** (Scheme 3), activated by [PhNMe_2_H][B(C_6_F_5_)_4_], 1-decene formed trisubstituted C_20_ olefins, and the yield of 9-methylenenonadecane was only 30% [52,53]. Tens of Group 4 metallocenes were studied in the coordination oligomerization of α-olefins (Scheme 3, Section 3); however, dimer fractions were the main reaction products only for the first dozen, **1**–**12**. 

The mechanism of the zirconocene-catalyzed dimerization of α-olefins remains unclear in terms of detail. On the surface, this mechanism is not very different from the conventional cationic mechanism, and the high selectivity of dimerization can be explained by the low value of the activation barrier of β-hydride transfer (or β-hydride elimination) after the insertion of the second molecule of monomer in comparison with the activation energy of the chain propagation. However, this interpretation does not match the results of the catalytic experiments. As far back as the 1990s [37,38], Christoffers and Bergman demonstrated that chloride is an indispensable component of the catalyst system that provides the high selectivity of dimerization; the special role of Zr–Cl bonding was also proposed by Hessen et al. [54]. Such an effect was confirmed experimentally by an increase in the selectivity of zirconocene/MAO-catalyzed dimerization in the presence of R_2_AlCl [40,55,56], and by the formation of oligomers when Cp_2_ZrMe_2_/B(C_6_F_5_)_3_ was used [57]. To explain the experimental facts, Nifant’ev et al. proposed a binuclear Zr–Al mechanistic concept [40,41] involving the Zr-(μ-Cl)(μ-H)AlR_2_ catalytic species (Scheme 2c). The early studies of the interaction of **1** with TIBA and HAl(^i^Bu)_2_ [58,59,60,61] were the experimental basis of this concept. 

**Figure 1:**
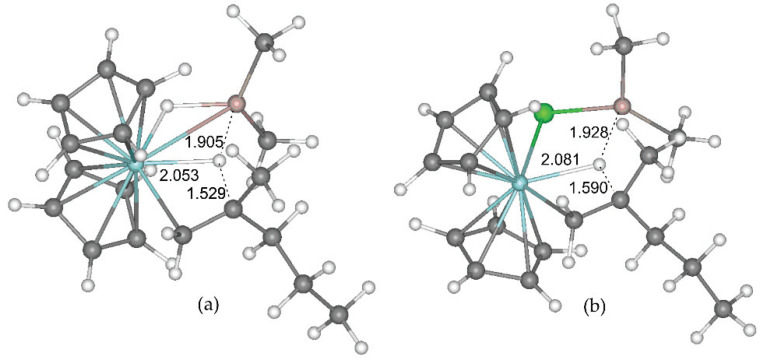
Cooperative effect in Zr–Al transition states of the β-hydride elimination stage in the framework of the Zr–Al binuclear mechanism [62]. Copyright (2019) MDPI.

Recently, this Zr–Al binuclear mechanism was studied theoretically for propylene oligomerization [62]. Using the quantum chemical modeling at the M-06x/DGDZVP level of the density functional theory (DFT), the qualitative difference between mononuclear and binuclear mechanisms (for [(η^5^-C_5_H_5_)_2_Zr–Alkyl]^+^ and [(η^5^-C_5_H_5_)_2_Zr–Alkyl(R_2_AlX)]^+^ species, respectively), was demonstrated. Without R_2_AlX coordination (mononuclear mechanism), oligomerization was found to be a preferable reaction pathway. In binuclear complexes (X = H, Cl), the formation of vinylidene dimers had been greatly facilitated by an explicit Zr–Al cooperative effect (Figure 1).

### 2.2. Dimerization of α-Olefins Catalyzed by Other Complexes of Transition and Rare-Earth Metals

**Scheme 4:**
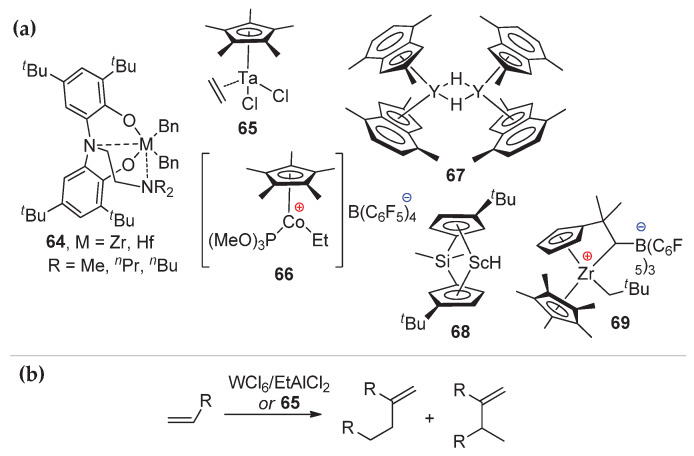
Other metal complexes that are active in the selective oligomerization of α-olefins (**a**); formation of vinylidene olefins in the presence of W and Ta complexes (**b**).

A number of complexes—namely, WCl_6_/Et_2_AlCl [63], zirconium and hafnium adducts with tetradentate ligand **64** [64], half-sandwich complexes of Ta **65** [65,66] and Co **66** [67], a bis-indenyl complex of Y **67** [68], a zwitter-ionic zirconium complex **69** [54], and a sandwich hydride complex of Sc **68** [69]—were studied in the coordination dimerization of α-olefins (Scheme 4a). In the presence of a WCl_6_-based catalyst, a mixture of vinylidene olefins was formed (Scheme 4b); similar mixtures were obtained in the presence of **65**. The half-sandwich complex **66** catalyzed the formation of dimers containing significant amounts of linear olefins. The sandwich complexes of rare-earth metals **67** and **68**, as well as the zwitter-ionic complex **69** demonstrated moderate catalytic activity and high selectivity in the formation of the single reaction product; however, the catalytic performance of these complexes dramatically decreased over time (TON ~ 100). Among the complexes mentioned above, only **64Hf** (R = nBu) was capable of beating zirconocenes on the criteria of selectivity and catalytic productivity; however, its stability was much lower. 

## 3. Coordination Oligomerization of α-Olefins

### 3.1. Common Aspects of the Coordination Oligomerization of α-Olefins

**Scheme 5:**
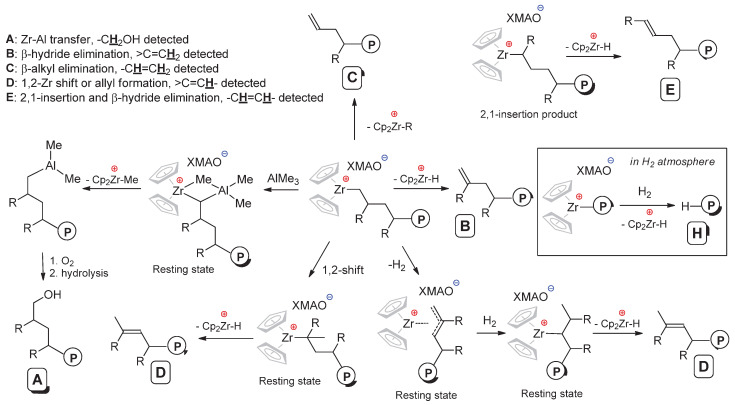
Traditional mechanisms of chain release in α-olefin polymerization with a formation of characteristic chain-end groups (routes A–E) and saturated polyolefin species (route H) [72,73,80].

Different types of transition metal complex were studied in the coordination oligomerization of α-olefins. Traditional Ziegler–Natta catalysts demonstrated low activity [70,71] and will not be discussed in our review. Group 4 metal sandwich complexes, metallocenes, are the most efficient and most extensively researched catalysts for this process. Scheme 5 illustrates the main and side reactions observed during metallocene-catalyzed oligomerization. The degree of polymerization, *DP*_n_, is related to a variety of factors, such as the molecular structure of metallocene, type and quantity of the activator, reaction temperature, and presence of the molecular hydrogen. In the heyday of metallocene catalysis (1990s–early 2000s), the zirconocene-catalyzed polymerization of higher α-olefins was largely viewed as a model process for the study of the mechanism of chain propagation and chain release [72,73,74,75,76,77,78,79]. 

Usually, the zirconocene-catalyzed oligomerization and polymerization of higher α-olefins was studied in the laboratory using the activation of LZrCl_2_ pre-catalysts by MAO with extremely high Al/Zr ratios (10^3^–10^4^), or the activation of LZrMe_2_ pre-catalysts by perfluoroaryl borates. Many of the zirconocenes under these conditions catalyzed the formation of high MW polyolefins (*M*_n_ ~10^4^–10^5^ Da); however, several complexes were efficient in the synthesis of oligomers (see below, Section 3.2). High Al_MAO_/Zr ratios facilitate the formation of Zr–Al alkyl complexes [81,82,83,84,85] that are unable to perform monomer coordination. Such complexes are intermediates of the chain release via Zr–Al transfer [86,87,88]. At the same time, the practice of using high Al_MAO_/Zr ratios in the laboratory was due to lthe ow effectiveness of MAO as an alkylation agent; the Al_MAO_/Zr ratios of 10^2^ MAO were found to be insufficient for the activation of bis-indenyl and bis-fluorenyl complexes [89]. In 2017, Nifant’ev et al. proposed a two-stage activation method for a wide array of LZrCl_2_ complexes. This method was based on the reaction of LZrCl_2_ with TIBA (formation of Zr–Al alkyl-hydrides) followed by the reaction with ~10 eq. of aMAO; dozens of zirconocene dichlorides were studied in such industrially important conditions [40,80,90,91,92] (see Section 3.3). In contrast to dimerization, the activation of zirconocenes by perfluoroaryl borates was successfully used in the oligomerization of higher α-olefins (see Section 3.4). The formulas of the metallocenes studied in the oligomerization of α-olefins are presented in Scheme 3.

### 3.2. Metallocene-Catalyzed Oligomerization of α-Olefins and Activation by 10^2^–10^3^ eq. MAO

Dozens of scientific articles and patents have been devoted to the oligomerization of higher α-olefins, catalyzed by Zr and Hf sandwich complexes and activated by a large excess of MAO (Table 1). The main target of the studies, namely, the synthesis of Group 4 oil base stocks, required the use of specific types of product characteristic such as the kinematic viscosity at a given temperature, KV^t^ (KV^100^ are given in Table 1), and viscosity index, VI, as an alternative to the degree of polymerization, *DP*_n_, or oligomer distribution.

The degree of oligomerization, *DP*_n_, depends on the structure of the metallocene pre-catalyst, Al_MAO_/Zr ratio, and reaction conditions. Unsubstituted zirconocene dichloride **1** and monosubstituted and disubstituted zirconocenes catalyzed the formation of the mixtures of lower oligomers. The presence of bulky alkyl or aryl substituents in the cyclopentadienyl rings resulted in decreasing activities of metallocenes and an increased content of higher oligomers; increasing the number of alkyl substituents entailed the same effect. The early results in the study of the oligomerization of α-olefins in the presence of zirconocenes **1** and **8** at Al_MAO_/Zr ~ 200 [47] were complemented substantially by Nifant’ev et al. [91], who established that under these conditions, the products of side reactions (2-alkenes and alkanes) typically exceeded 10 wt %. Therefore, bis-cyclopentadienyl complexes at high Al_MAO_/Zr ratios have poor prospects for the oligomerization of α-olefins. 

The derivatives of substituted indenes and fluorenes demonstrated more promising catalytic properties. High yields of the oligomer fractions were obtained for metallocenes **53**, **56** [98], and **61** [93]. For these complexes, the degree of oligomerization, *DP*_n_, can be affected by the hydrogen pressure and reaction temperature with no reduction in the yield of the oligomer fraction.

In order to conclude this section on metallocene-catalyzed oligomerization at high Al_MAO_/Zr ratios, it is important to note the publication of Jiang et al. [101], which was a thorough study of the microstructure of 1-butene/1-dodecene copolymers, obtained in the presence of (η^5^-C_5_Me_4_H)_2_ZrCl_2_, activated by 100–500 eq. of MAO. Detailed investigations of the kinetics and mechanisms of the formation of α-olefin oligomers in the presence of (η_5_-C_5_H_4_-*n*-Bu)_2_ZrCl_2_ [102] and (η^5^-C_5_H_5_)_2_ZrCl_2_ [103] after activation by 10^2^–10^3^ eq. of MAO have also been of particular interest.

### 3.3. Zirconocene-Catalyzed Oligomerization of α-Olefins at Low Al_MAO_/Zr Ratios

A systematic study of the zirconocene-catalyzed oligomerization of α-olefins at low Al_MAO_/Zr ratios was started in recent years. With the use of the method of the two-stage activation of LZrCl_2_ (with TIBA and then MAO, see Section 3.1), Nifant’ev et al. studied the catalytic performance of a wide range of zirconocenes [80,90,91,92]. The results of these studies are presented in Table 2.

During the research, the side reactions of the isomerization and reduction of the starting α-olefins were studied. In was established that in some cases, the content of 2-alkenes in the reaction products can reach values of 20% and higher (for example, for zirconocene **36** [90]). The formation of 2-alkenes is a greatly underestimated problem of the metallocene-catalyzed oligomerization of α-olefins; the isomerization of the starting monomers to inert internal olefins seriously diminishes the yields of oligomers, thus generally devaluing the method of metallocene-catalyzed oligomerization in the production of polyolefin oils and lubricants.

The feasibility of the ligand design is a substantial advantage of the zirconocene catalysis; the inherently high stability of the bis(η^5^-cyclopentadienyl)Zr fragment allows for a fruitful search of the catalyst without the drawbacks of the unnecessary wasting of the starting α-olefins.

**Figure 2:**
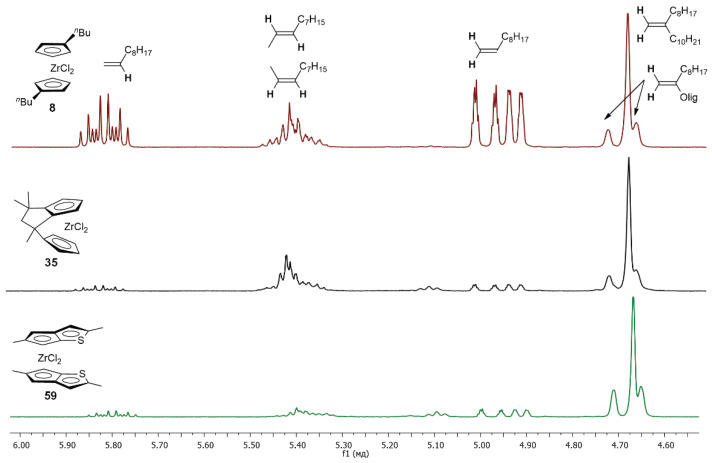
Fragments of ^1^H NMR spectra of the reaction mixtures of 1-decene oligomerization (2 h, 100 °C), catalyzed by zirconocenes **8**, **35**, and **59**, activated by 10 eq. of MMAO-12. Reprinted with permission from [91]. Copyright (2018) Elsevier B.V.

Figure 2 illustrates this idea; activation by 10 eq. of modified methylalumoxane MMAO-12, bis-cyclopentadienyl complex **8** was clearly inferior to that by *ansa*-zirconocene **35** in terms of catalytic activity, but both zirconocenes **8** and **35** were less active and selective than zirconocene **59** [91]. Using **59**, Nifant’ev et al. separated oligomers of 1-hexene, 1-octene, and 1-decene for *DP*_n_ = 2–5 and proved the homogeneity of their structure [91]; similar results were reported later by Mi et al., who used the less active zirconium complexes **1**, **2**, **10**, and **50** [104]. The complex **59** is a representative of metallocenes containing heterocycle-fused η^5^-cyclopentadienyl fragments, “heterocenes”. In the early 2000s, such complexes were extensively studied by the chemists of the Basell Polyolefins and Exxon Mobil companies in close cooperation with M.V. Lomonosov Moscow University in the polymerization of ethylene and propylene [105,106,107,108]. To date, heterocenes are considered as the most promising single-site oligomerization catalysts (see Section 4.2 and Section 5).

### 3.4. Zirconocene-Catalyzed Oligomerization of α-Olefins, Activated by Perfluoroaryl Borates

To avoid the use of a large excess of organoaluminium in the activation of metallocenes, MAO can be replaced with perfluoroaryl borates. This method, proposed by Marks et al. [109,110,111], has been successfully used in the oligomerization of α-olefins (Table 3).

The activation by perfluoroalkyl borates, apparently, resulted in the formation of more electrophilic separate ion pairs, which caused the possibility of side processes unusual for zirconocene/MAO catalysts. The ^1^H NMR analysis of the end-groups of 1-decene oligomers, obtained in the presence of zirconocenes **32**–**34**, clearly demonstrated the product β-hydride’s elimination from the secondary Zr–alkyl complexes [112]. The formation of such complexes does not affect the carbon skeleton of the oligomers; however, it inevitably results in the slowing down of the oligomerization. A substantially more important process—namely, the β-elimination of the *n*-hexyl fragment (Scheme 5, reaction pathway C)—was detected in the oligomerization of 1-octene catalyzed by bis(indenyl) hafnium complex **54** [73]. The products of this side reaction represent allyl-terminated oligooctenes that are able to react with the formation of long-chain branched polymers.

Experiments on the oligomerization of 1-decene in the presence of the **47-Me**/[PhNHMe_2_][B(C_6_F_5_)_4_] catalytic system, performed by the chemists of Total company [125], deserve special notice. The content of the C_30_ fraction in the reaction products reached a value of 70%, comparing favorably with those in BF_3_-catalyzed oligomerization. 

In addition to MAO and perfluoroaryl borates, fluorinated aluminosilicate, in combination with TIBA, was successfully used in the zirconocene-catalyzed oligomerization of α-olefins [97] (Table 4).

### 3.5. Post-Metallocene Catalysts in the Oligomerization of α-Olefins

**Scheme 6:**
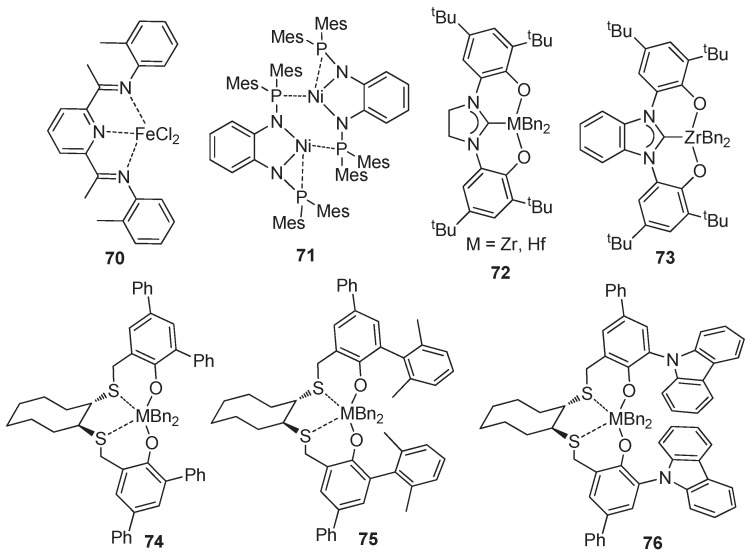
Post-metallocene pre-catalysts studied in the non-selective coordination oligomerization of α-olefins.

**Scheme 7:**
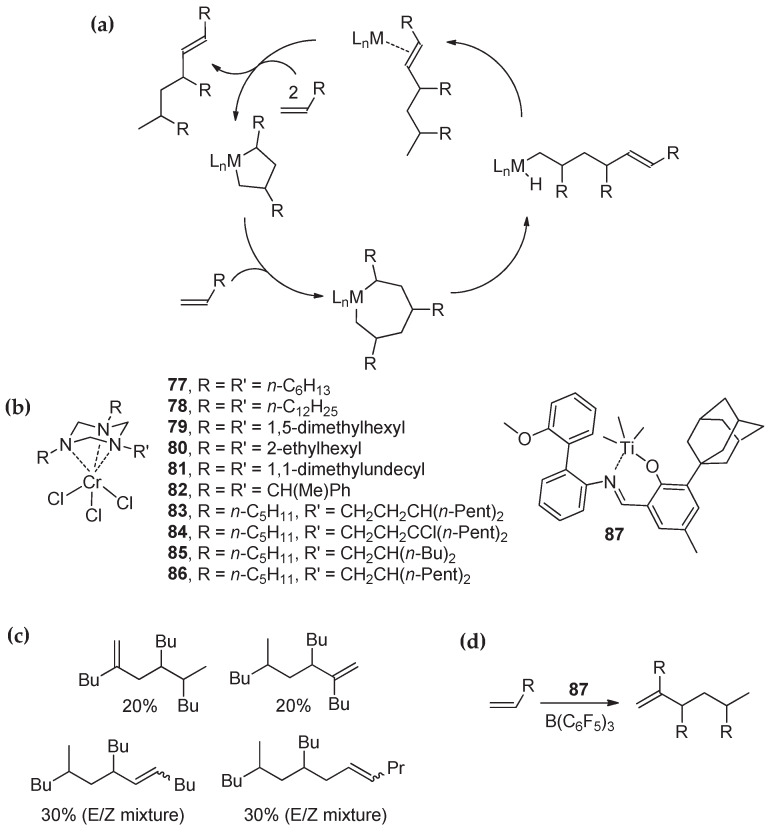
(**a**) Metallacyclic mechanism of the coordination trimerization of α-olefins; (**b**) Precatalysts of trimerization; (**c**) Product distribution for the trimerization of 1-hexene, catalyzed by **77**/MAO [142]; (**d**) The main product of the trimerization of α-olefins, catalyzed by **87 [146]**.

Post-metallocene catalysts that have been intensively and fruitfully studied in the polymerization and oligomerization of ethylene and propylene [126,127,128,129,130,131,132,133,134] were of limited use in the oligomerization of higher α-olefins. The Group 8 metal complexes **70** [135] and **71** [136] (Scheme 6) were inferior to zirconocenes in terms of catalytic activity and, therefore, are of no interest for practical applications. The Group 4 post-metallocenes have greater potential in selective oligomerization. The carbene zirconium complex **72** with bulky phenolate fragments (Scheme 7) in the presence of [Ph_3_C][B(C_6_F_5_)_4_] demonstrated moderate activity in the non-selective oligomerization of 1-hexene; however, activation by [PhNHMe_2_][B(C_6_F_5_)_4_] resulted in the formation of a trimerization product with ~77% yield [137]. At the same time, the benzimidazole analog **73** catalyzed non-selective oligomerization regardless of the type of activator [138]. Zirconium complexes with [OSSO]-type ligands **74**, **75** [139], and **76** [140] (Scheme 6) in the presence of dried modified methylaluminoxane (dMMAO) catalyzed the formation of lower 1-hexene oligomers with excellent vinylidene selectivity. Apparently, the mechanism of the oligomerization catalyzed by **72**–**76** is similar to the mechanism of zirconocene-catalyzed oligomerization.

The metallacyclic mechanism of the coordination oligomerization of α-olefins (Scheme 7a) [130,141] is highly attractive regarding the selectivity of the formation of the trimer fraction. This mechanism is considered proven for chromium (III) complexes **77**–**82** of tridentate cyclic ligands (Scheme 7b) [142,143,144,145]. In the presence of 100 eq. of MAO, the complexes **77** and **78** catalyzed the trimerization of 1-hexene with high selectivity; dimers and tetramers were found in less than 1%, and TONs ~10^3^ were detected at room temperature [142]. The structures and ratios of the major isomers in the C_18_ fraction are presented in Scheme 7c. One year later [143], Wasserscheid et al. studied the trimerization of 1-decene and 1-dodecene, catalyzed by a series of the complexes **79**–**82** with different substituents at N atoms; the complex **80** with 2-ethylhexyl substituents demonstrated the best catalytic performance in terms of trimerization selectivity and productivity. The impact of the steric bulk of the alkyl substituent R’ on trimerization selectivity was studied in 2016 by Cohon and Köhn [144] for complexes **83**–**86** (Scheme 7b). The ratios of the products of trimerization of 1-hexene are presented in Table 5.

The metallacyclic mechanism was also proposed for the Ti (IV) complex **87** [146]. In the presence of B(C_6_F_5_)_3_, this complex demonstrated more than 95% selectivity in the trimerization of 1-pentene, 1-hexene, and 1-decene with TONs ~350 (C_5_, C_6_) and 100 (C_10_). Among the trimers produced, ca. 85% were one regioisomer (Scheme 7d). The major olefin product is proposed to form by a tail-to-tail coupling, followed by 1,2-insertion and selective β-hydride elimination. 

The theoretical aspects of three possible Cosse–Arlman, Green–Rooney, and metallacyclic mechanisms of α-olefin oligomerization were studied theoretically by Copéret et al. [147]; the findings of this original article warrant further experimental studies. 

Note that the Group 8 metal complexes demonstrated high efficiency in the oligomerization of ethylene [148,149,150] and norbornene [149,150,151]. The promising results in the selective oligomerization of propylene [152] and oligomerization/polymerization of 1-butene [153,154] and higher α-olefins [153,155,156,157,158] allow the consideration of such complexes as prospective post-metallocene oligomerization catalysts if the problem of moderate productivity could be solved.

## 4. The Use of Methylenealkanes

As shown above (see Section 2.1), methylenealkanes can be easily obtained by the zirconocene-catalyzed dimerization of α-olefins. The yields of linear α-olefin dimers typically exceed values of 90% in the event that an efficient pre-catalyst (i.e., **6**, Scheme 2b) is used [40]. In addition, methylenealkanes are unavoidable by-products of α-olefin oligomerization (see Section 3). The problem of the utilization of α-olefin dimers is of great relevance and importance [33,41,159]. 

**Figure 3:**
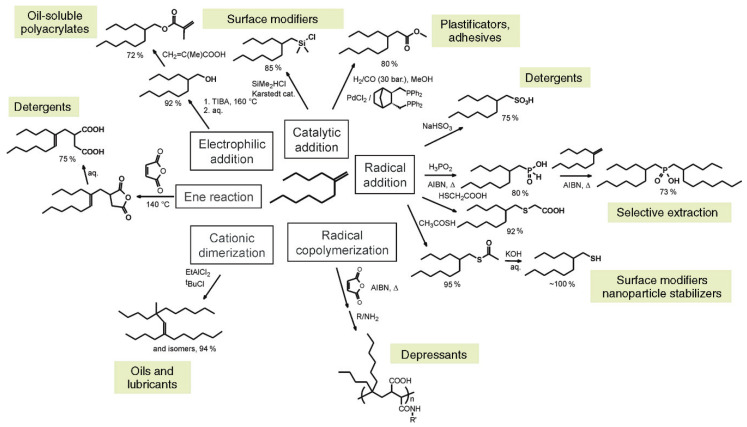
5-methyleneundecane derivatives and their prospective applications. Reprinted with permission from [41]. Copyright (2017) De Gruyter.

Methylenealkanes are close structural analogs of isobutylene. The presence of the reactive C=C bond and a substantial difference in the environments of these unsaturated carbon atoms allows the consideration of methylenealkanes as prospective starting compounds for the regioselective synthesis of amphiphilic organic molecules and polymers. A number of possible directions for the synthetic use of methylenealkanes were demonstrated by us with the example of 5-methyleneundecane [41] (Figure 3). Below, we will refer to some of the notable examples of the use of methylenealkanes in the synthesis of organic compounds and polymers.

### 4.1. Free Radical Addition to Methylenealkanes

**Scheme 8:**
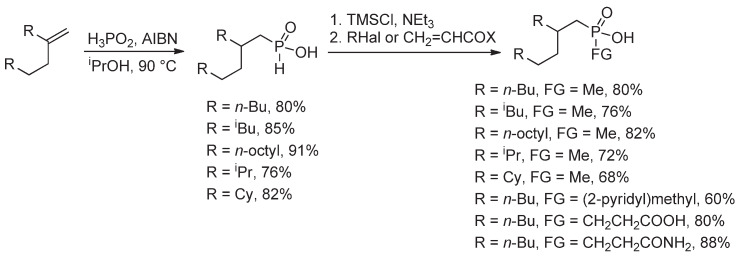
Synthesis of alkylphosphinic and disubstituted phosphonic acids [161].

The ease of free radical addition of MeC(O)SH using the method developed by Klotz et al. [160] was demonstrated on the example of 5-methyleneundecane (Figure 3) [41]. The closely related free-radical hydrophosphinylation of methylenealkanes was studied by Nifant’ev et al. in order to obtain hydrolytically stable extractants of rare-earth metals [161] (Scheme 8). Branched alkylphosphinic acids, obtained by the hydrophosphinylation of methylenealkanes, also demonstrated promising anti-wear properties [162].

### 4.2. Free Radical Polymerization of Methylenealkanes

**Scheme 9:**
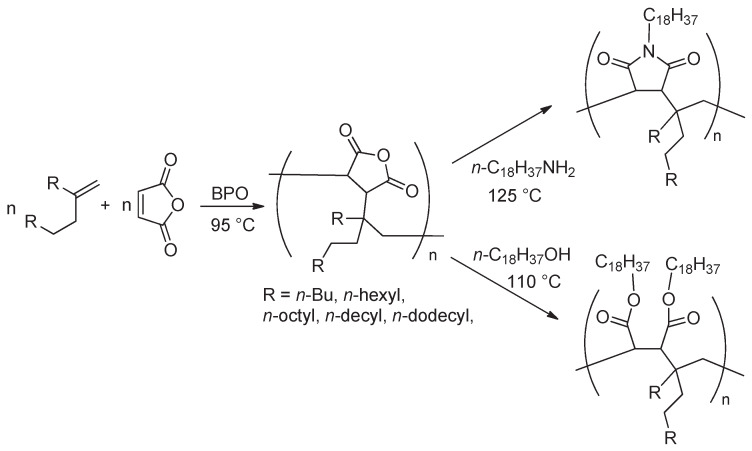
Synthesis and modification of alternating copolymers of methylenealkanes and maleic anhydride [164].

**Figure 4:**
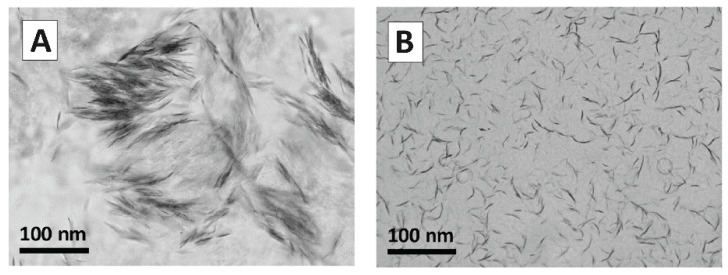
Morphology of paraffin crystals in the absence (**A**) and in the presence (**B**) of the pour point depressant, the octadecanol-modified copolymer of maleic anhydride and 9-methylenenonadecane [164].

The ability of methylenealkanes to form copolymers with polar vinyl monomers was demonstrated by Yamago et al. with the example of the organotellurium-mediated living radical polymerization (TERP) of 6-methyleneundecane with acrylates; the tendency to form alternating copolymers was demonstrated [163]. Later, Nifant’ev et al. studied the copolymerization of a series of α-olefin dimers with maleic anhydride [164]. In copolymerization experiments, performed in hydrocarbon media at 80–100 °C, azobisisobutyronitrile (AIBN)- or benzoyl peroxide (BPO)-initiated reactions resulted in the formation of copolymers with 1:1 comonomer ratios (Scheme 9), which would suggest an alternating nature of the reaction products. The post-modification of the copolymers obtained by the reactions with higher linear amines and alcohols (Scheme 9) resulted in copolymers with promising pour point depressant characteristics (Figure 4).

**Figure 5:**
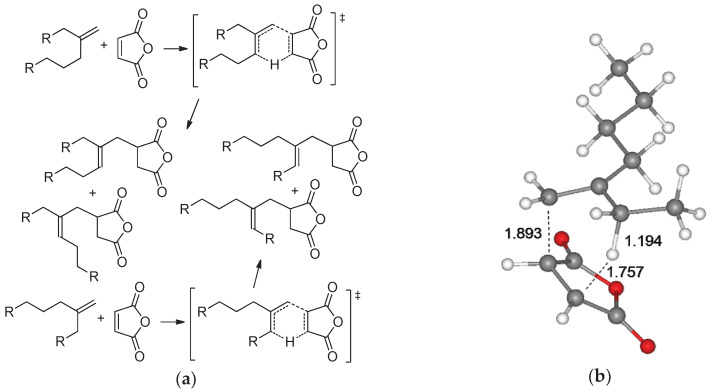
(**a**) The Alder-ene reaction between maleic anhydride (MA) and methylenealkanes; (**b**) Optimized geometry of the transition state of the Alder-ene reaction between MA and 3-methyleneheptane [165].

The DFT modeling of copolymerization of olefins with maleic anhydride (MA) [165] confirmed the preference of the alternating reaction pathway for methylenealkanes. The possible Alder-ene side reaction (Figure 5a) was also studied; the results of calculations demonstrated that this thermally induced process cannot complete with alternating polymerization under the reaction conditions due to the relatively high level of the free energy of the corresponding transition state (Figure 5b). At elevated temperatures (180–200 °C), this reaction proceeded within 4–6 h with good yields [165,166]; the Alder-ene adducts were used as a starting compounds in the synthesis of bis-succinimide friction modifiers for transmission fluids [166].

### 4.3. Epoxydation and Related Reactions

**Scheme 10:**
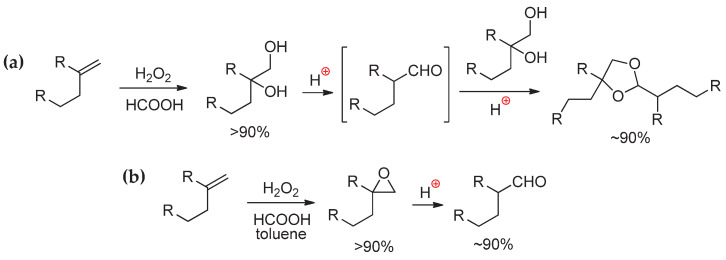
Epoxidation of methylenealkanes with a formation of (**a**) 1,2-diols or (**b**) oxiranes; and related reactions.

The reaction of methylenealkanes with H_2_O_2_/HCOOH resulted in the formation of the corresponding 1,2-diols with good yields (Scheme 10a) [167]. The acid-catalyzed rearrangement of the diols to the corresponding aldehydes, followed by the reaction with 1,2-diol, yielded branched acetals (Scheme 10a) [167]. Using toluene as a solvent, the reaction was stopped at the stage of the oxirane [168,169]; the acid-catalyzed rearrangement of the latter in the presence of H_5_Mo_12_O_41_P yielded the corresponding aldehyde (Scheme 10b) [168]. The first reaction sequence was the subject of the study of Harvey et al. [170] that represents a perfect example of neglect of the patent sources.

### 4.4. Methylenealkanes as Alkylating Reagents

**Scheme 11:**
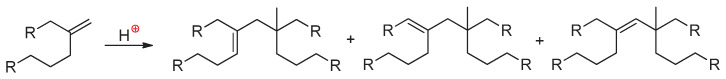
Electrophilic dimerization of α-olefin dimers, methylenealkanes [47,91,171].

In contrast with the acid-catalyzed oligomerization of linear α-olefins, accompanied by rearrangements with the formation of a large number of isomeric products, methylenealkanes in the presence of acids form products with the same carbon skeleton (Scheme 11). Kissin and Schwab achieved 90% conversions of 5-methyleneundecane after 5 h of the reaction at 60 °C with 87% “dimer of dimer” selectivity using silica-supported EtAlCl_2_ [47]. The C_40_ fraction obtained by the dimerization of 9-methylenenonadecane (dimer of 1-decene) had promising viscosity characteristics (KV^100^ of 6.4 cSt and VI of 147) [47]. The product of the hydrogenolysis of this compound represents a promising low-viscosity PAO base [171]. Nifant’ev et al. proposed a more efficient catalytic system (1 mol % of *tert*-BuCl and EtAlCl_2_) that allowed the achievement of 98% conversions of 1-hexene, 1-octene, and 1-decene dimers after 2 h of the reaction at –30 °C [91]. Methylenealkanes were also used in the electrophilic alkylation of diphenylamine, catalyzed by acid-treated clays or ionic liquids [172].

### 4.5. Catalytic Transformations of Methylenealkanes

**Scheme 12:**
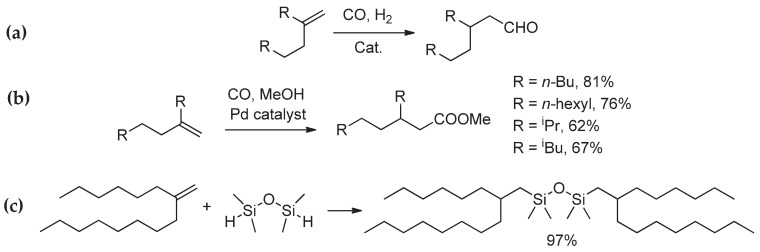
Catalytic transformations of methylenealkanes: (**a**) Hydrofirmylation [173,175]; (**b**) Methoxycarbonylation [176]; (**c**) Hydrosilylation [178].

The hydroformylation of 9-methylenenonadecane in the presence of 0.01 mol % of (Ph_3_P)_3_Rh(CO)H resulted in the formation of the corresponding aldehyde (Scheme 12a) with a 76% yield [173]. A phosphine-modified cobalt catalyst was less efficient (66% yield) [174]. The best results, a 92% yield with 99% selectivity, were obtained using a (acac)Rh(CO)_2_/PPh_3_ catalyst [175]. The closely related methoxycarbonylation of methylenealkanes (Scheme 12b) was accompanied by the isomerization of the starting hydrocarbons; the rational design of the Pd/diphosphine catalyst had made it possible to reach average yields of the branched methyl esters [176]. The esters obtained were used in the synthesis of Group 5 oil base stocks with promising viscosity characteristics [177].

Catalytic hydrosilylation was another potentially significant process that allowed the obtaining of highly branched disiloxane with promising viscosity characteristics [178] (Scheme 12c). The same products can be clearly obtained by the hydrolysis of the branched chlorosilanes easily accessible via the hydrosilylation by Me_2_SiHCl, catalyzed by a Karstedt catalyst [41] (see Figure 3).

## 5. Oils and Lubricants Based on Coordination Oligomers of α-Olefins

**Figure 6:**
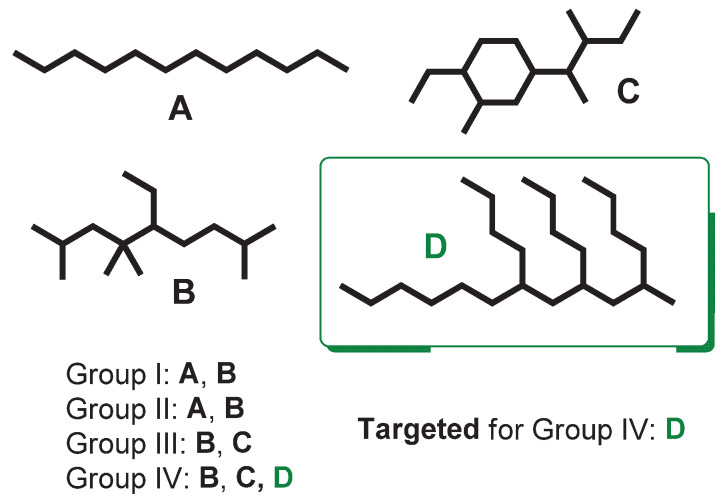
Main structural types of hydrocarbons—components of oils. Reprinted with permission from [91]. Copyright (2018) Elsevier B.V.

As was demonstrated previously, the viscosity properties such as *VI* and pour point (*PP*) of poly-α-olefin (PAO) oil base stocks depend strongly on the architecture of the constituent hydrocarbons [16,17,179,180]. Linear hydrocarbons, petroleum waxes, (Figure 6, A), which are present in large amounts in Group I and II oils, have high *PP*s. Consequently, these oils have limitations for their use since they cannot provide secure low-temperature engine start and transmission performance. Group III oil base hydrocarbons, which contain compounds with short branches (Figure 6, B) and cycloalkanes (Figure 6, C), also have relatively high *PP*s and low-temperature viscosity values. Long-chain branched hydrocarbons (Figure 6, D) are characterized by low *PP*s and high *Vi*s and therefore represent the most prospective group of hydrocarbons for use as high-grade bases of engine oils and transmission fluids.

**Scheme 13:**
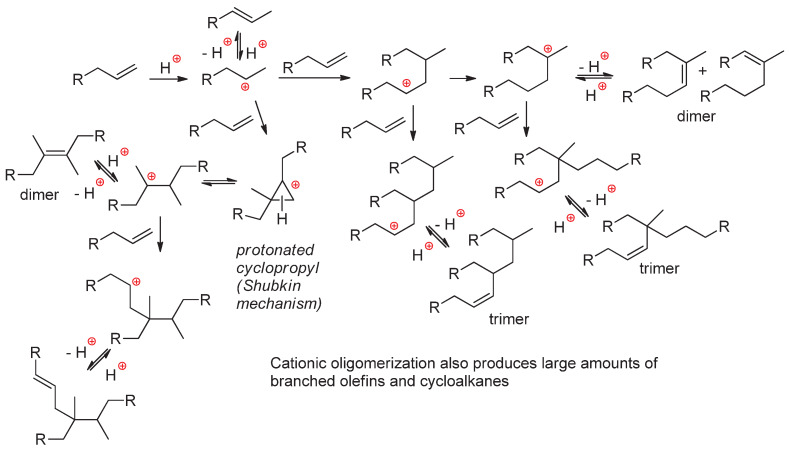
Rearrangements in the cationic oligomerization of α-olefins [14,15].

**Figure 7:**
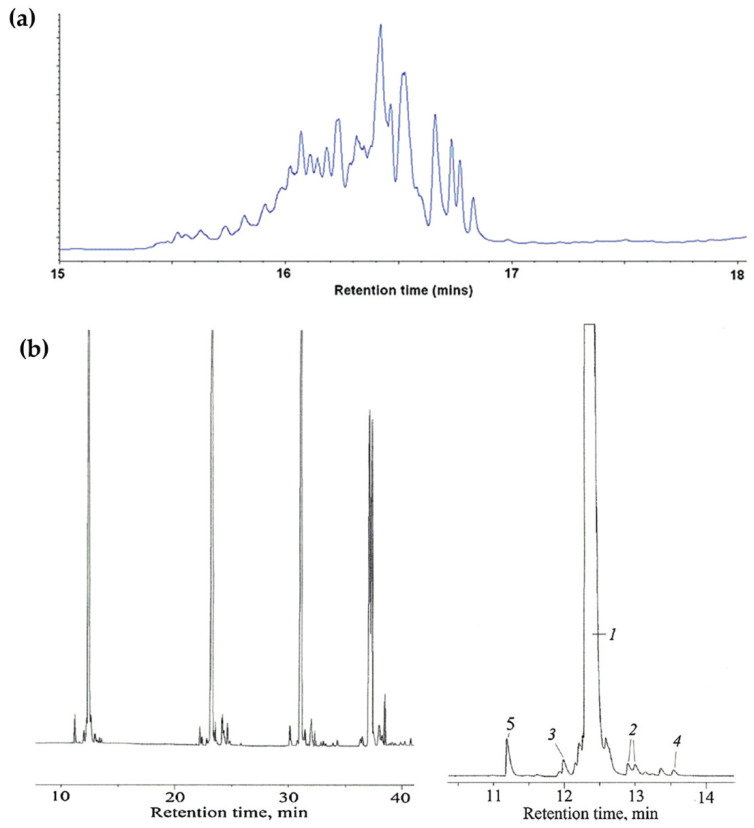
(**a**) Gas chromatogram showing peaks for C_20_ olefin isomers during acid-catalyzed 1-decene oligomerization. Reprinted with permission from [15]. Copyright (2012) Wiley-VCH Verlag GmbH & Co; (**b**) Gas chromatorgams (C_12_–C_30_ range, left) and dimer range (right) of 1-hexene oligomers produced with the **8**/MAO catalyst at 70 °C. Reprinted with permission from [103]. Copyright (2019) Elsevier B. V.

As mentioned in the Introduction section, cationic oligomerization is accompanied by a huge number of rearrangements, including peculiar reactions proposed by Shubkin [14] and studied later by Gee et al. [15] (Scheme 13). The products of metallocene-catalyzed oligomerization are vinylidene-type α-olefin oligomers with uniform molecular structures (Scheme 1a). A gas chromatogram of the C_20_ fraction of the products of the cationic oligomerization of 1-decene (Figure 7a) [15] confirms the complexity of the process with the formation of large number of reaction products; the difference between this grim picture and the gas chromatogram of the products of zirconocene-catalyzed oligomerization (Figure 7b) [103] clearly establishes the prospects of “metallocene” oligomers in terms of the structural homogeneity.

**Figure 8:**
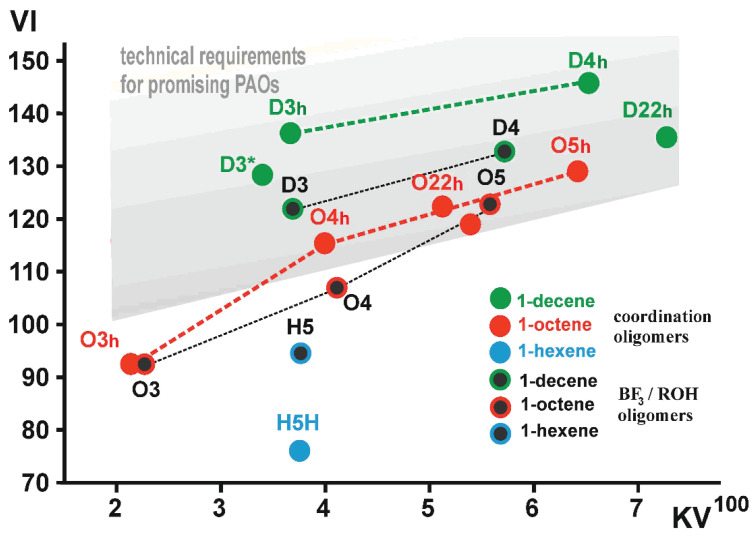
Viscosity characteristics of 1-decene (green), 1-octene (red), and 1-hexene (blue) oligomers obtained by coordination polymerization [91] and in the presence of BF_3_–ROH [16]. D3*: decene trimer obtained using [EBTHI]ZrCl_2_–TIBA–perfluoroborate [52]. The range of technical requirements for promising poly-α-olefin oils (PAOs) was limited to the segment corresponding to VI = 100 for KV^100^ = 2 and VI = 120 for KV^100^ = 6. Reprinted with permission from [91]. Copyright (2018) Elsevier B. V.

The transformation of the α-olefin oligomers to PAO base stocks necessitates catalytic hydrogenation. Electrophilic oligomerization led to the partial formation of tetra-substituted olefins (Scheme 13); the presence of >C=C< fragments significantly hampers the complete hydrogenolysis of double bonds. The ease of the hydrogenation is an additional benefit of the coordination α-olefin oligomers. The viscosity characteristics of the α-olefin oligomers obtained using electrophilic and metallocene catalysts and hygrogenated oligomers (PAO basestocks) are given in Table 6 and illustrated by Figure 8 [91]. These data clearly demonstrate that 1-hexene oligomers have little or no value for use as PAO base stocks due to low viscosity indices and high pour point values. The oligomers of 1-octene prepared by the zirconocene-catalyzed reaction have medium viscosity and outperform the 1-octene oligomers obtained in the presence of BF_3_–ROH according to viscosity indices, while possessing the same low-temperature kinematic viscosity. The oligomers of 1-decene synthesized by the zirconocene-catalyzed process significantly outperform the electrophilic oligomers obtained in the presence of BF_3_–ROH and can be considered as base stocks for modern PAOs.

The above pertains to oligomers obtained by the zirconocene-catalyzed process (Scheme 1a); however, as mentioned in Section 3.5, coordination oligomerization can proceed by a metallacyclic mechanism with the formation of products with different molecular structures (Scheme 8). The comparison of the viscosity characteristics of 1-decene trimers obtained by the zirconocene-catalyzed reaction and a metallacyclic process (Cr catalysts **79**, **80**, **82**) suggests that metallocene catalysis is preferable for the production of PAO oil base stocks. 

## 6. Conclusions

Thus, it can be concluded that the zirconocene-catalyzed oligomerization of higher α-olefins represents a flexible and resource-efficient method for the synthesis of methylenealkanes (vinylidene dimers of α-olefins, >90% yields with >98% selectivities) and oligomers with a given *DP*_n_. A broader view of the importance of the molecular structure and molecular design that emerged over recent years resulted in the development of advanced “metallocene” technologies for poly-α-olefin oils and lubricants by leading petrochemical companies such as Exxon, Idemitsu, and Mobil. The further progress in this field is related to the creation of the novel metallocene catalysts in order to achieve enhanced thermal stability, catalytic productivity, and selectivity in the synthesis of the desired oligomer fractions. Our recent research on 1-octene oligomerization and polymerization [80] further strongly suggests the high potential of “heterocenes” as a new generation of single-site catalysts of α-olefin oligomerization. We have obtained early results that are extremely promising.

The problem of the utilization of methylenealkanes, which are imminent side products of metallocene-catalyzed oligomerization, is still relevant. The fundamental difference between methylenealkanes and isobutylene consists of the ability of methylenealkanes to isomerize with the formation of more stable branched olefins with >C=CH– fragments. The research and development of the catalysts and processes without such isomerization is an actual affront to researchers’ professionalism.

## Figures and Tables

**Table 1:** Metallocene-catalyzed oligomerization of α-olefins at high Al/Zr ratios

Cat.	Mon.	[Mon]/[Zr]	[Al]/ [Zr]	T, °C	H_2_, bar	TOF, h^−1^	*DP*_n_	*KV* ^100 1^	*VI* ^2^	Additional Remarks	Ref.
							or Oligomer Distribution for *DP*_n_ 2,3,4,5				
**1**	C_6_	1.3 × 10^5^	4000	50	–	5.9 × 10^4^	4.9	–	–	–	[45,46]
	C_6_	2 × 10^3^	200	60	–	–	% olig. 45 (2):20 (3–5), res. 21 ^3^				[91]
	C_8_	2 × 10^3^	200	60	–	–	% olig. 45 (2):25 (3–5), res. 15				[91]
	C_10_	1.0 × 10^5^	1000	40	–	1.1 × 10^4^	–	41	196	–	[93]
	C_10_	1.0 × 10^5^	1000	80	–	3.2 × 10^4^	–	2.5	181	–	[93]
	C_10_	3.0 × 10^4^	30	50	1	2.9 × 10^4^	% olig. 42:11:17:5, res. 35				[51]
	C_10_	5.0 × 10^4^	300	70	–	9.2 × 10^3^	–	17	167	24% of dimer	[49]
	C_10_	5.0 × 10^4^	300	110	–	3.7 × 10^3^	–	5.9	152	55% of dimer	[49]
	C_14_	1.6 × 10^3^	530	40	–	1.1 × 10^3^	% olig. 42:25:16:8, res. 6				[50]
	C_14_	1.6 × 10^3^	530	60	–	>2 × 10^3^	% olig. 60:23:8:4, res. 5				[50]
**3**	C_10_	1.0 × 10^5^	1000	40	–	1.1 × 10^4^	–	2460	344	–	[93]
**7**	C_6_	1.3 × 10^5^	4000	50	–	9.4 × 10^4^	5.7	–	–	–	[45,46]
**8**	C_6_	1.3 × 10^5^	4000	50	–	2.0 × 10^4^	3.9	–	–		[45,46]
	C_6_	2 × 10^3^	200	60	–	-	% olig. 15 (2):30 (3–5), res. 41				[91]
	C_8_	500	200	60	–	-	% olig. 31 (2):41 (3–5), res. 16				[91]
	C_10_	1.0 × 10^5^	1000	90	–	6.3 × 10^4^	–	2.3	163		[93]
	C_10_	3.0 × 10^4^	30	50	1	2.7 × 10^4^	% olig. 47:26:11:4, res. 12				[51]
	C_10_	3.0 × 10^4^	100	50	1	>3 × 10^4^	% olig. 44:27:11:5, res. 13				[51]
	C_10_	3.0 × 10^4^	300	50	1	2.8 × 10^4^	% olig. 54:28:9:3, res. 6				[51]
	C_10_	3.0 × 10^4^	600	50	1	2.9 × 10^4^	% olig. 27:28:16:9, res. 20				[51]
	C_10_	5.0 × 10^4^	300	110	–	6.0 × 10^3^	–	6.7	156	49% of dimer	[49]
	C_10_	5.0 × 10^4^	300	110	–	6.0 × 10^3^	–	6.7	156	49% of dimer	[49]
**9**	C_10_	2.7 × 10^3^	340	50	–	>3 × 10^3^	% olig. 43:24:11:5, res. 17				[50]
	C_10_	3.0 × 10^4^	30	50	1	1.6 × 10^4^	% olig. 28:12:8:6, res. 46				[51]
	C_10_	5.0 × 10^4^	300	110	–	6.0 × 10^3^	–	5.7	152	50% of dimer	[49]
**13**	C_6_	1.3 × 10^5^	4000	50	–	3.2 × 10^4^	14	-	-		[45,46]
**14**	C_10_	5.0 × 10^4^	300	110	–	6.1 × 10^3^	–	8.2	159	40% of dimer	[49]
**19**	C_10_	3 × 10^4^	30	50	1	1.5 × 10^4^	% olig. 25:24:10:6, res. 25				[51]
**20**	C_10_	5.0 × 10^4^	300	70	–	1.1 × 10^4^	–	61	190	6% of dimer	[49]
	C_10_	5.0 × 10^4^	300	110	–	9.8 × 10^3^	–	17	162	15% of dimer	[49]
**23**	C_10_	3.0 × 10^4^	30	50	1	8 × 10^3^	% olig. 13:5:4:3, res. 75				[51]
**24**	C_6_	1.3 × 10^5^	4000	50	–	7.5 × 10^4^	14	–	–		[45,46]
**25**	C_6_	1.3 × 10^5^	4000	50	–	3.9 × 10^4^	45	–	–		[45,46]
**26**	C_6_	1.3 × 10^5^	4000	50	–	6.5 × 10^4^	83	–	–		[45,46]
	C_10_	5.0 × 10^4^	300	70	–	1.2 × 10^4^	-	154	–	1% of dimer	[49]
**28**, X = Cl	C_6_	1.3 × 10^5^	4000	50	–	3.4 × 10^4^	19	–	–		[45,46]
	C_10_	5.0 × 10^4^	300	70	–	2.3 × 10^3^	–	115	224	5% of dimer	[49]
**37**	C_6_	1.0 × 10^3^	500	25	–	630	~3	49% of dimer			[94]
**38**	C_10_	1.5 × 10^5^	1000	80	–	8.3 × 10^4^	26	101	–		[95]
	C_10_	1.5 × 10^5^	1000	80	5	8.2 × 10^4^	14	28	–		[95]
	C_10_	1.5 × 10^5^	1000	80	15	1.3 × 10^5^	12	32	–		[95]
**41**	C_10_	1.0 × 10^5^	250	100	–	1.1 × 10^4^	72	–	–		[96]
**42**	C_8_	1.7 × 10^5^	1000	25	40		63	831	–		[97]
**44**	C_10_	1.5 × 10^5^	1000	80	–	7.4 × 10^4^	27	143	–		[95]
	C_10_	1.5 × 10^5^	1000	80	5	1.2 × 10^5^	17	71	–		[95]
	C_10_	1.5 × 10^5^	1000	80	15	1.9 × 10^5^	19	86	–		[95]
**47**	C_10_	1.0 × 10^5^	1000	40	–	1.0 × 10^5^	–	702	296		[93]
**48**	C_10_	2.6 × 10^5^	600	80	6.9	611	–	11	211		[98]
**51**	C_10_	1.3 × 10^5^	500	80	13.8	8.3 × 10^4^	–	112	208		[98]
**52**	C_10_	1.0 × 10^5^	1000	40	–	5.0 × 10^4^	–	2460	344		[93]
	C_10_	2.6 × 10^5^	200	100	13.8	8.9 × 10^4^	21	116	214		[98]
**53**	C_10_	2.6 × 10^5^	250	100	6.9	2.6 × 10^5^	19	94	213		[98]
	C_10_	2.6 × 10^5^	250	90	–	5.6 × 10^4^	133	1227	–		[98]
	C_10_	2.6 × 10^5^	250	90	1.7	1.0 × 10^5^	57	453	–		[98]
	C_10_	2.6 × 10^5^	250	90	3.4	2.6 × 10^5^	40	333	–		[98]
	C_10_	2.6 × 10^5^	250	90	6.9	2.6 × 10^5^	19	94	–		[98]
	C_10_	2.6 × 10^5^	250	90	13.8	2.1 × 10^5^	12	39	179		[98]
**56**	C_10_	4.2 × 10^5^	260	80	13.8	1.4 × 10^5^	21	25	183		[98]
**57**	C_8_	2.5 × 10^5^	1000	65	–	1.4 × 10^5^	23	139	192		[99]
	C_8_	2.5 × 10^5^	1000	65	8	2.2 × 10^5^	18	43	168		[99]
**60**	C_8_	2.0 × 10^5^	1000	25	–	1.3 × 10^5^	83	1119	–		[97]
**61**	C_10_	1.0 × 10^5^	1000	40	–	1.0 × 10^5^	–	2460	344		[93]
	C_10_	3.0 × 10^5^	1000	70	–	2.4 × 10^5^	64	635	282		[93]
	C_10_	3.0 × 10^5^	1000	150	–	1.6 × 10^5^	–	58	195		[93]
	C_10_	3.0 × 10^5^	500	70	–	2.3 × 10^5^	–	1134	307		[93]
	C_10_	3.0 × 10^5^	250	70	–	1.7 × 10^5^	–	1308	314		[93]
	C_10_	3.0 × 10^5^	1000	70	0.5	1.5 × 10^5^	–	1074	308		[93]
	C_10_	3.0 × 10^5^	1000	70	1	2.1 × 10^5^	–	863	296		[93]
	C_10_	3.0 × 10^5^	1000	70	2	2.0 × 10^5^	–	722	288		[93]
	C_10_	3.0 × 10^5^	1000	70	13	2.4 × 10^5^	–	512	271		[93]
	C_6_	3.0 × 10^5^	1000	90	–	1.8 × 10^5^	–	2862	251		[93]
	C_8_	3.0 × 10^5^	1000	90	–	2.0 × 10^5^	–	888	276		[93]
	C_10_	3.0 × 10^5^	1000	90	–	1.6 × 10^5^	–	515	272		[93]
	C_12_	3.0 × 10^5^	1000	90	–	1.3 × 10^5^	–	402	264		[93]
**62**	C_10_	7.0 × 10^5^	1500	63	8	1.6 × 10^5^	15	37	177		[100]

^1^ Kinematic viscosity at 100 °C. ^2^ Viscosity index. ^3^ In % by weight, *DP*_n_ in the brackets, res.—residue, higher oligomers.

**Table 2:** Zirconocene-catalyzed oligomerization of α-olefins at low Al/Zr ratios

Cat.	Mon.	[Mon]/[Zr]	Al_TIBA_/Zr	Al_MAO_ /Zr	T, °C	H_2_, bar	Conv. (h)	*DP*_n_	Additional Remarks	Ref.
								or Oligomer Distribution		
**1**	C_6_	2 × 10^3^	20	10	60	–	97 (4)	% olig. 85 (2):12 (3–5), res. 1 ^1^		[91]
**8**	C_6_	2 × 10^3^	20	10	60	–	85 (4)	% olig. 35 (2):36 (3–5), res. 20		[91]
	C_6_	2 × 10^3^	20	10	60	1	99 (4)	% olig. 50 (2):31 (3–5), res. 11		[91]
	C_8_	2 × 10^3^	20	10	60	–	83 (4)	% olig. 40 (2):40 (3–5), res. 11		[91]
	C_10_	2 × 10^3^	20	10	60	–	72 (4)	% olig. 38 (2):35 (3–5), res. 17		[91]
	C_10_	2 × 10^3^	20	10	100	–	69 (4)	% olig. 40 (2):36 (3–5), res. 10		[91]
**10**	C_6_	2 × 10^3^	20	10	60	–	16 (4)	% olig. 40 (2):30 (3–5), res. 19		[91]
**18**	C_6_	2 × 10^3^	20	10	60	–	92 (4)	% olig. 35 (2):31 (3–5), res. 31		[91]
**22**	C_6_	2 × 10^3^	20	10	60	–	72 (4)	% olig. 8 (2):34 (3–5), res. 55		[91]
**26**	C_6_	4 × 10^3^	80	40	60	–	90 (4)	38		[92]
**30**	C_6_	2 × 10^3^	20	10	60	–	89 (4)	44		[91]
**31**	C_6_	4 × 10^3^	80	40	60	–	75 (4)	190		[92]
**35**	C_6_	2 × 10^3^	20	10	60	–	98 (4)	% olig. 41 (2):28 (3–5), res. 24		[91]
	C_8_	2 ×·10^3^	20	10	60	–	98 (4)	% olig. 36 (2):32 (3–5), res. 29		[91]
	C_10_	2 × 10^3^	20	10	60	–	98 (4)	% olig. 40 (2):33 (3–5), res. 35		[91]
	C_10_	2 × 10^3^	20	10	100	–	98 (4)	% olig. 41 (2):34 (3–5), res. 19		[91]
**36**	C_6_	2·× 10^3^	20	10	60	–	98 (4)	% olig. 40 (2):30 (3–5), res. 11		[90]
**41**	C_6_	2 × 10^3^	20	10	60	–	95 (4)	% olig. 22 (2):32 (3–5), res. 39		[91]
**42**	C_6_	2 × 10^3^	20	10	60	–	94 (4)	% olig. 20 (2):31 (3–5), res. 40		[91]
**45**	C_6_	2·× 10^3^	20	10	60	–	88 (4)	25		[91]
	C_6_	4 × 10^3^	80	40	60	–	76 (4)	25		[92]
**46**	C_6_	2 × 10^3^	20	10	60	–	94 (4)	108		[91]
**47**	C_6_	2 × 10^3^	20	10	60	–	92 (4)	37		[91]
	C_6_	4 × 10^3^	80	40	60	–	94 (4)	35		[92]
**50**	C_6_	4 × 10^3^	80	40	60	–	95 (4)	108		[92]
**55**	C_6_	4 × 10^3^	80	40	60	–	95 (4)	68		[92]
**59**	C_6_	2 × 10^3^	20	10	60	–	100 (4)	% olig. 9 (2):17 (3–5), res. 72		[91]
	C_6_	2 × 10^3^	20	10	60	1	100 (4)	% olig. 13 (2):24 (3–5), res. 57		[91]
	C_8_	2 × 10^3^	20	10	60	–	100 (4)	% olig. 23 (2):30 (3–5), res. 45		[91]
	C_8_	2 × 10^3^	20	10	80	–	100 (4)	% olig. 28 (2):26 (3–5), res. 44		[91]
	C_8_	2 × 10^3^	20	10	100	–	100 (4)	% olig. 37 (2):34 (3–5), res. 27		[91]
	C_8_	2 × 10^3^	20	10	120	–	100 (4)	% olig. 44 (2):32 (3–5), res. 21		[91]
	C_8_	2 × 10^3^	20	10	100	1	100 (4)	% olig. 47 (2):34 (3–5), res. 8		[91]
	C_8_	2 × 10^3^	20	10	120	1	100 (4)	% olig. 51 (2):40 (3–5), res. 10		[91]
	C_10_	2 × 10^3^	20	10	100	–	93 (4)	% olig. 44 (2):45 (3–5), res. 7		[91]
	C_10_	2 × 10^3^	20	10	100	1	99 (4)	% olig. 45 (2):41 (3–5), res. 6		[91]
	C_10_	2 × 10^3^	20	10	120	1	99 (4)	% olig. 51 (2):33 (3–5), res. 10		[91]

^1^ The ratios of dimer and lower oligomer fractions, and residue.

**Table 3:** Zirconocene-catalyzed oligomerization of α-olefins, with activation by perfluoroaryl borates.

Cat.	Mon.	[Mon] /[Zr]	Al/Zr	T, °C	H_2_, bar	TOF, h^−1^	*DP*_n_	*KV* ^100^	*VI*	AlR_3_, Additional Remarks	Ref.
							or Oligomer Distribution for *DP*_n_ 2,3,4,5+				
**11, X = Me**	C_10_	5.2 × 10^5^	50	100	–	1.0·× 10^3^	27	103	–	Al(*n*-Oct)_3_, NB ^1^	[113]
	C_10_	5.2·× 10^5^	50	100	3	3.6·× 10^5^	23	11.3	–	Al(*n*-Oct)_3_, NB	[113]
**16, X = Me**	C_10_	5.2·× 10^5^	50	100	–	5.2·× 10^4^	10.6	4.5	–	Al(*n*-Oct)_3_, NB	[113]
	C_10_	5.2·× 10^5^	50	120	–	3.1·× 10^4^	8.8	3.4	–	Al(*n*-Oct)_3_, NB	[113]
	C_10_	5.2·× 10^5^	50	100	3.7	7.8·× 10^4^	9.5	3.7	–	Al(*n*-Oct)_3_, NB	[113]
	C_10_	5.2·× 10^5^	50	120	3	8.0·× 10^4^	8.3	3.1	–	Al(*n*-Oct)_3_, NB	[113]
**17, X = Me**	C_10_	5.2·× 10^5^	50	100	–	8.4·× 10^3^	18	10.2	–	Al(*n*-Oct)_3_, NB	[113]
	C_10_	5.2·× 10^5^	50	120	–	1.2·× 10^4^	11	4.9	–	Al(*n*-Oct)_3_, NB	[113]
	C_10_	5.2·× 10^5^	50	120	3	5.9·× 10^4^	10.6	4.5	–	Al(*n*-Oct)_3_, NB	[113]
**8**	C_10_	5.2·× 10^5^	50	100	–	6.3 × 10^4^	9.3	4.0	–	Al(*n*-Oct)_3_, NB	[113]
	C_10_	5.2·× 10^5^	50	120	–	3.9 × 10^4^	7.9	3.1	–	Al(*n*-Oct)_3_, NB	[113]
	C_10_	5.2·× 10^5^	50	100	3	7.4·× 10^4^	9.0	3.8	–	Al(*n*-Oct)_3_, NB	[113]
	C_10_	5.2·× 10^5^	50	120	3	8.8·× 10^4^	7.9	3.1	–	Al(*n*-Oct)_3_, NB	[113]
**20**	C_10_	5.2·× 10^5^	50	100	3	8.7·× 10^4^	7.9	3.0	–	Al(*n*-Oct)_3_, NB	[113]
	C_10_	5.2·× 10^5^	50	120	3	8.8·× 10^4^	7.5	2.8	–	Al(*n*-Oct)_3_, NB	[113]
**21, X = Me**	C_10_	8.5·× 10^4^	4	50	–	1.3·× 10^4^	-	96	199	Al(*n*-Oct)_3_, NB	[114]
	C_10_	8.5·× 10^4^	4	60	1	1.6·× 10^4^	-	44	180	Al(*n*-Oct)_3_, NB	[114]
	C_10_	8.5·× 10^4^	4	70	1	1.8·× 10^4^	-	20	179	Al(*n*-Oct)_3_, NB	[114]
	C_10_	8.5·× 10^4^	4	80	1	2.0·× 10^4^	-	11.0	163	Al(*n*-Oct)_3_, NB	[114]
	C_10_	8.5·× 10^4^	4	100	1	1.7·× 10^4^	-	8.0	161	Al(*n*-Oct)_3_, NB	[114]
	C_10_	8.5·× 10^4^	4	120	1	2.1·× 10^4^	-	5.0	151	Al(*n*-Oct)_3_, NB	[114]
	C_10_	5.2·× 10^5^	50	80	–	2.2·× 10^4^	13	5.9	–	Al(*n*-Oct)_3_, NB	[113]
	C_10_	5.2·× 10^5^	50	100	–	3.3 ×·10^4^	10	4.1	148	Al(*n*-Oct)_3_, NB	[113]
	C_10_	5.2·× 10^5^	50	120	–	3.3·× 10^4^	8.5	3.3	–	Al(*n*-Oct)_3_, NB	[113]
	C_10_	5.2·× 10^5^	50	100	3	1.2·× 10^5^	8.6	3.3	–	Al(*n*-Oct)_3_, NB	[113]
**26**	C_10_	1.2·× 10^5^	100	110	–	–	–	3.9	144	TIBA, NB	[115]
**27, X = Me**	C_10_	5.2·× 10^5^	50	100	–	7.2·× 10^3^	10	4.1	–	Al(*n*-Oct)_3_, NB	[113]
	C_10_	5.2·× 10^5^	50	120	–	1.8·× 10^4^	8.3	3.1	–	Al(*n*-Oct)_3_, NB	[113]
	C_10_	5.2·× 10^5^	50	100	3	1.1·× 10^5^	9.3	3.6	–	Al(*n*-Oct)_3_, NB	[113]
	C_10_	5.2·× 10^5^	50	120	3	3.1·× 10^4^	8.0	3.0	–	Al(*n*-Oct)_3_, NB	[113]
**28, X = Me**	C_10_	5.2·× 10^5^	50	100	3	2.0·× 10^4^	7.6	2.8	–	Al(*n*-Oct)_3_, NB	[113]
	C_10_	5.2·× 10^5^	50	120	3	1.4·× 10^4^	7.9	3.2	–	Al(*n*-Oct)_3_, NB	[113]
**32**	C_10_	2.6·× 10^5^	200	120	–	4.7·× 10^4^	9.6/7.0/4.0/2.6 + 64			TIBA, CB ^2^	[112]
**33**	C_8_	2.6·× 10^5^	200	120	–	1.9·× 10^5^	13.1/14.1/11.8/33.4			TIBA, CB	[112]
	C_10_	2.6·× 10^5^	200	120	–	1.5·× 10^5^	13.3/14.3/11.3/34.7			TIBA, CB	[112]
**34**	C_10_	2.6·× 10^5^	200	120	–	9.1·× 10^4^	17.0/18.4/13.9/24.6			TIBA, CB	[112]
**38**	C_10_	1.5·× 10^5^	200	120	+	1.2·× 10^5^	6.6	5.5	164	TIBA, NB	[95]
	C_10_	1.5·× 10^5^	200	140	+	1.2·× 10^5^	4.4	2.6	140	TIBA, NB	[95]
**39, X = Me**	C_10_	2.0·× 10^5^	20	120	–	~2·× 10^5^	~3	C30 3.4 C40 9.3	128 158	Al(*n*-Oct)_3_, NB	[52,53]
	C_10_	8.9·× 10^4^	0.01	60	–	7.3·× 10^4^	32	169	225	Al(*n*-Oct)_3_, NB	[116]
	C_10_	8.9·× 10^4^	0.01	80	–	8.4·× 10^4^	14	26.5	169	Al(*n*-Oct)_3_, NB	[116]
	C_10_	8.9·× 10^4^	0.01	100	–	8.8·× 10^4^	9.3	11.2	150	Al(*n*-Oct)_3_, NB	[116]
**40**	C_10_	3.6·× 10^5^	>100	70	–	3.1·× 10^5^	-	18.5	164	TIBA, CB	[117]
	C_10_	3.6·× 10^5^	>100	100	–	2.0·× 10^5^	-	8.9	153	TIBA, NB	[117]
	C_10_	3.6·× 10^5^	>100	100	–	2.5·× 10^5^	-	14.6	158	TIBA, CB	[117]
**48**	C_10_	3.5·× 10^5^	>100	50	–	1.5·× 10^5^	226	3715	379	TIBA, NB	[117]
	C_10_	3.5·× 10^5^	>100	84	–	2.9·× 10^5^	80	724	291	TIBA, NB	[117]
	C_10_	3.5·× 10^5^	>100	100	–	2.3·× 10^5^	64	363	250	TIBA, NB	[117]
**50**	C_10_	2–4·× 10^5^	20-200	60–100	1–10	1.0·× 10^5^	–	253–586	247–281	TIBA, NB	[118]
**58**	C_8_/C_12_	1.0·× 10^5^	10	100	0.5	~1·× 10^5^	15–35	39–140	175–212	TIBA, NB	[119]
	C_8_/C_10_/C_12_	1.0·× 10^5^	10	100	0.5	~1·× 10^5^	15–30	40–120	179–205	TIBA, NB	[120]
	C_8_/C_10_	1.0·× 10^5^	10	100	0.5	~1·× 10^5^	19–32	45–109	175–204	TIBA, NB	[121]
**61**	C_10_	4.4·× 10^5^	10	80	2	3.6·× 10^5^	-	834	304	TIBA, NB	[122]
	C_10_	4.4·× 10^5^	10	105	2	2.4·× 10^5^	76	622	289	TIBA, NB	[123,124]
	C_10_	4.4·× 10^5^	10	110	2	3.2·× 10^5^	71	558	280	TIBA, NB	[122]
	C_10_	4.4·× 10^5^	10	120	2	2.4·× 10^5^	63	434	270	TIBA, NB	[122]
	C_10_	4.4·× 10^5^	10	130	2	2.4·× 10^5^	57	377	266	TIBA, NB	[122]

^1^ NB: [PhNMe_2_H][B(C_6_F_5_)_4_]. ^2^ CB: [CPh_3_][B(C_6_F_5_)_4_].

**Table 4:** Oligomerization of 1-octene, catalyzed by zirconium complexes, activated by triisobutylalumnium (TIBA) and fluorinated aluminosilicate [97].

Cat.	[Mon]/[Zr]	T, °C	TOF, h^–1^	*KV* ^100^	*VI*
**1**	1.1 × 10^5^	90	3.1 × 10^3^	8.9	211
**8**	5.6 × 10^5^	105	4.1 × 10^4^	45	175
**9**	3.1 × 10^5^	110	3.4 × 10^4^	62	186
**15**	1.5 × 10^6^	70	1.3 × 10^5^	130	222
**29**	5.5 × 10^5^	90	2.5 × 10^4^	8.3	157
**42**	4.3 × 10^5^	120	3.1 × 10^4^	159	214
**43**	4.4 × 10^5^	120	3.6 × 10^4^	132	200
**45**	1.1 × 10^5^	90	9.0 × 10^3^	23	169
**47**	4.2 × 10^5^	115	4.3 × 10^4^	136	210
**49**	1.0 × 10^5^	100	5.2 × 10^3^	10.3	194

**Table 5:**
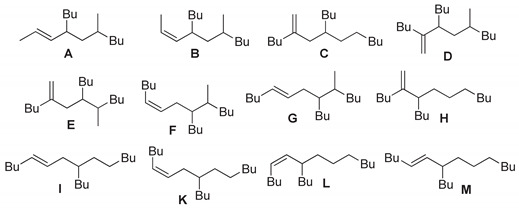
The regioisomers produced in the trimerization of 1-hexene with a range of catalysts [144].

Cat.	Regioisomer Abundance (%)											
	A	B	C	D	E	F	G	H	I	K	L	M
**83**	39.9	22.4	11.8	16.5	3.6	2.0	0.9	0.4	0.2	1.2	0.4	0.7
**84**	38.5	19.2	15..5	15.6	3.7	1.9	0.8	0.6	0.2	1.5	0.4	1.4
**85**	46.9	15.3	3.0	19.7	8.3	3.1	1.5	0.3	0.2	0.7	0.3	0.7
**86**	36.9	28.8	1.6	19.8	9.1	2.2	0.7	0.1	<0.1	0.4	<0.1	0.3

**Table 6:** Viscosity characteristics of α-olefin oligomers.^1^

No. of C Atoms	Oligomer	*KV*^−40^, sSt	*KV*^40^, sSt	*KV*^100^, sSt	*VI*	*PP*, °C	Ref.
**12**	**H2H**	9.00	1.28	–	–	−73	[91]
**16**	**O2**	–	2.6	–	–		[91]
**16**	**O2H**	53.1	2.82	–	–	−43	[91]
**18**	**H3**		3.12				[91]
**18**	**H3h**	167.2	3.57	–	–	−94	[91]
**18**	**H3h** (BF_3_ catalyst)	165	3.8	1.4	–	<−55	[16]
**20**	**D2**		4.55	1.7	14		[91]
**20**	**D2h**		5.30	–	14	−7	[91]
**24**	**H4**		7.55	2.10	62		[91]
**24**	**H4h**	1335	8.93	2.28	46	−79	[91]
**24**	**H4h** (BF_3_ catalyst)	1780	9.8	2.6	94		[16]
**24**	**H22h**	3030	12.4	2.72	27	−71	[91]
**24**	**O3**		6.5	2.06	114		[91]
**24**	**O3h**	552.1	7.56	2.20	92	−86	[91]
**24**	**O3H** (BF_3_ catalyst)	580	8.0	2.3	92	<−55	[16]
**30**	**H5**		17.55	3.6	76		[91]
**30**	**H5h**	6798	19.2	3.8	76	−67	[91]
**30**	**H5h** (BF_3_ catalyst)	7850	18.1	3.8	96		[16]
**30**	**D3**		14.61	3.65	140		[91]
**30**	**D3h** (catalyst **79**)		12.2	3.2	126		[143]
**30**	**D3h** (catalyst **80**)		13.0	3.3	131		[143]
**30**	**D3h** (catalyst **82**)		12.1	3.2	137		[143]
**30**	**D3h**	1897	15.05	3.70	137	−75	[91]
**30**	**D3h** [EBTHI]Zr - borate		13.5	3.39	128		[52]
**30**	**D3h** (BF_3_ catalyst)	2070	15.6	3.7	122	<−55	[16]
**32**	**O4**		13.94	3.44	125		[91]
**32**	**O4h**	3135	18.4	4.0	115	−74	[91]
**32**	**O4h** (BF_3_ catalyst)	4750	20.0	4.1	106	<−55	[16]
**32**	**O22**		28.1	5.4	130		[91]
**32**	**O22h**	6374	29.4	5.4	119	−68	[91]
**40**	**O5**		32.9	6.05	132		[91]
**40**	**O5h**	11651	36.2	6.4	129	−68	[91]
**40**	**O5h** (BF_3_ catalyst)	10225	30.9	5.6	124		[16]
**40**	**D4**		31.3	6.03	142		[91]
**40**	**D4h**	8631	34.5	6.52	145	−66	[91]
**30**	**D4h** (BF_3_ catalyst)	7475	29.0	5.7	141		[16]
**40**	**D22**		29.43	5.90	150		[91]
**40**	**D22h**	15615	42.0	7.25	136	−52	[91]

^1^ Abbreviations used: **H**—1-hexene, **O**—1-octene, **D**—1-decene oligomers; **2**–**5**—*DP*_n_ value; **22**—dimer of dimer; **h**—hydrogenated.
